# Factors associated with trajectories of bone marrow lesions over 4 years: data from the Osteoarthritis Initiative

**DOI:** 10.1007/s00256-024-04579-6

**Published:** 2024-01-20

**Authors:** Ziyuan Shen, Xiaoyue Zhang, Yining Wang, Rui Zhu, Liru Ge, Guoqi Cai

**Affiliations:** 1 Department of Epidemiology and Biostatistics, School of Public Health, Anhui Medical University, Hefei, 230032 Anhui China; 2 Menzies Institute for Medical Research, University of Tasmania, Hobart, TAS 7000 Australia

**Keywords:** Bone marrow lesions, Osteoarthritis, Risk factors, Trajectory

## Abstract

**Objective:**

To identify bone marrow lesion (BML) trajectories over 4 years and their demographic and structural predictors in middle-aged and older adults with or at increased risk of knee osteoarthritis (OA).

**Methods:**

A total of 614 participants (mean age 61 years, 62% female) from the Osteoarthritis Initiative cohort (OAI) were included. BMLs in 15 anatomical locations of the knee were measured annually from baseline to 4 years using the Magnetic Resonance Imaging Osteoarthritis Knee Score (MOAKS) method. BML trajectories were determined using latent class mixed models (LCMMs). Multinomial logistic regression was used to examine baseline characteristics that predicted BML trajectories.

**Results:**

Three distinct BML trajectories were identified: “Mild-stable BMLs” (25.9%), “Moderate-stable BMLs” (66.4%), and “Rapid-rise BMLs” (7.7%). Compared to the “Mild-stable BMLs” trajectory, current smokers were more likely to be in the “Moderate-stable BMLs” (odds ratio [OR] 2.089, *P* < 0.001) and “Rapid-rise” (OR 2.462, *P* < 0.001) trajectories. Moreover, female sex and meniscal tears were associated with an increased risk of being in the “Rapid-rise BMLs” trajectory (OR 2.023 to 2.504, *P* < 0.05). Participants who had higher education levels and drank more alcohol were more likely to be in the “Rapid-rise BMLs” trajectory (OR 1.624 to 3.178, *P* < 0.05) and less likely to be in the “Moderate-stable BMLs” trajectory (OR 0.668 to 0.674, *P* < 0.05).

**Conclusions:**

During the 4-year follow-up, most participants had relatively stable BMLs, few had enlarged BMLs, and no trajectory of decreased BMLs was identified. Sociodemographic factors, lifestyle, and knee structural pathology play roles in predicting distinct BML trajectories.

**Supplementary Information:**

The online version contains supplementary material available at 10.1007/s00256-024-04579-6.

## Introduction

Osteoarthritis (OA) is a complex chronic disease mainly characterized by joint pain, affecting millions of people worldwide, especially in the elderly [1]. Bone marrow lesions (BMLs) are common structural abnormalities seen on magnetic resonance images (MRIs) that have been found to be associated with pain and cartilage degeneration and an increased risk of knee replacement [2–6].

Unlike inreversible structural changes such as joint space width and cartilage volume, BMLs can enlarge or shrink over time [7]. In a longitudinal study of 217 patients with clinical knee OA, BMLs enlarged or remained stable in 99% of participants over 30 months [8]. Foong et al. found that during an 8-year period, 79% of 198 participants experienced an increase or stable size of BMLs, and 52% of participants who did not have BMLs at baseline developed incident BMLs [9]. However, there are also studies showing that the majority of pre-existing BMLs shrank over 3 to 30 months [10, 11]. Importantly, the enlargement and shrinkage of BMLs may occur in several weeks and therefore could be a sensitive treatment target [11]. Although change in BMLs is an important surrogate marker for evaluating OA progression and even treatment effects, there is currently no study to assess the long-term trajectories of BMLs. Trajectory analysis allows for the exploration of patterns and trends in change in BMLs over multiple time points, providing more insights into the variation of BMLs. Moreover, previous studies have shown that age, weight, knee alignment, and meniscal pathology were associated with the risk of BMLs in the knee [7, 12–14], but factors related to the trajectories of BMLs are unknown. Therefore, we aimed to identify BMLs trajectories and their predictors in middle-aged and older adults with or at increased risk of knee OA.

## Methods

### Study design and participants

The report of this study followed the Strengthening the Reporting of Observational Studies in Epidemiology (STROBE) statement [15]. We utilized data from the Osteoarthritis Initiative (OAI), a multi-center, longitudinal, prospective observational study (https://nda.nih.gov/oai/). The OAI study included adults aged 45 to 79 years who either had knee osteoarthritis or were at increased risk of developing it. The risk factors for inclusion were older age (> 45 years), frequent knee symptoms, regular use of medications for knee symptoms, being overweight, a history of knee injury or surgery, a family history of OA, the presence of Heberden’s nodes, and engaging in activities that involve repetitive knee bending [16]. Participants were not eligible if they had inflammatory arthritis, bilateral end-stage knee osteoarthritis, bilateral knee replacement surgeries, or contraindications to 3 T magnetic resonance imaging. Ethics approvals were obtained from the institutional review board at each of the four clinical centers that recruited OAI participants. All participants provided written informed consent. Participants included in this study were from a sub-study of the OAI that evaluated the prevalence and development of MRI detected lesions (project 63), which included cases with incident radiographic OA and age-, sex-, and BMI-matched controls [17].

### Assessment of BMLs

The magnetic resonance imaging (MRI) exams of knees were read using the MOAKS (MRI Osteoarthritis Knee Score) scoring method [17]. Annual assessments of BMLs from baseline to 4 years were conducted in 15 anatomical locations by the sagittal and coronal IW TSE series, the sagittal 3D DESS WE, and the axial and coronal multiplanar reformats (MPRs) of the DESS series. The 15 locations were as follows: femur medial anterior (trochlear), femur lateral anterior (trochlear), femur medial central, femur lateral central, femur medial posterior, femur lateral posterior, tibia sub-spinous, tibia medial anterior, tibia lateral anterior, tibia medial central, tibia lateral central, tibia medial posterior, tibia lateral posterior, patella medial, and patella lateral. The MOAKS ranges from 0 to 3 for each subregion: 0 indicates no BML presence; 1 indicates BML involvement of less than 25% of the subregion’s area; 2 corresponds to BML involvement between 25 and 50% of the subregion’s area; and 3 indicates BML involvement of greater than 50% of the subregion’s area [18]. The total BMLs size was the sum of 15 anatomical positions, with possible scores ranging from 0 to 45.

### Covariates

Covariates that may be associated with the trajectories of BMLs were selected based on previous literature [19–24]. They included the following variables: age (year), gender (male, female), height, weight, education level (Less than or equal to high school, some college or college graduate, and some graduate school or graduate degree), marital status (married and unmarried/widowed/divorced), race (White, Black or African Americans, and other), alcohol consumption (none, < 1/week, 1–7/week, and > 7/week), smoking status (never, current, and former), history of knee surgery (other than knee replacement), history of knee injury and physical activity (the Physical Activity Scale for the Elderly) [25], Charlson comorbidity score [26], joint space narrowing (JSN) grades, and any meniscal tears. JSN was assessed for the medial and lateral tibiofemoral compartment according to the Osteoarthritis Research Society International atlas [27], ranging from grade 0 to grade 3 (i.e., 0 = normal, 1 = mild change, 2 = moderate change, 3 = severe change). The most severe JSN in the medial and lateral compartments was used as the JSN grade. Additionally, considering that weight change over time might impact BML, we included the 4-year weight change in our analysis. This change was calculated by subtracting the baseline weight from the weight recorded in the fourth year. Meniscal tears were assessed using the semi-quantitative MOAKS scoring method, with each of the three anatomical sub-regions (i.e., anterior horn, body, posterior horn) scored from 0 to 6. A meniscal tear was defined as a total MOAKS score of ≥ 1. Knee alignment was not included as a covariate because of its high proportion of missingness (73% missing).

### Statistical analysis

Baseline characteristics were presented as mean (standard deviation (SD)) and *n* (%). Analysis of variance and chi-square test were utilized for the comparison of different trajectory groups.

The latent class mixed models (LCMMs) were fitted using the R ‘lcmm’ package [28]. We tested models with different numbers and forms (linear, quadratic, and beta) of trajectories to determine the optimal trajectories [28, 29]. Beta is an optional family parameter from the family of Beta cumulative distribution functions in the model that describes the trend of trajectories over time. Moreover, the “linear” option specifies a linear link function leading to a standard linear mixed model, and the “Quadratic” models add a squared term to capture curved trends. The optimal model was selected based on goodness of fit indices including Akaike information criterion (AIC) [30] and Bayesian information criterion (BIC) [31], with smaller AIC and BIC values indicating a better model fit. The mean posterior probabilities (PP) and average PP were calculated, with a value of > 70% indicating a good fit of the identified trajectories [32, 33]. Meanwhile, each trajectory group of BMLs was required to contain at least 5% of the total participants to ensure further data analysis, which may have limited trajectories with few participants being identified, such as trajectories with decreasing or fluctuating BMLs [34].

Multinomial logistic regression analysis was used to examine factors predicting BML trajectories. The odds ratio (*OR*) and 95% confidence interval (95% *CI*) were calculated. Multiple imputation with chained equations (MICE) was adopted to account for missing data on baseline covariates, assuming missing at random. Five imputations were conducted using complete variables and non-missing values of imputed variables.

All statistical analyses were performed by R software (version 4.2.3; http://www.Rproject.org). Statistical significance was set at a *P* value of < 0.05 (two-tailed).

## Results

### Participants

A total of 614 participants were included in this study. The baseline characteristics are presented in Table 1. The mean (SD) age was 61.0 (9.0) years and 62.1% were female. There were 449 (73.1%) participants who had BMLs at baseline.

**Table 1:** Baseline characteristics of participants

Covariates	Overall (*n* = 614)	Mild-stable BMLs (*n* = 159)	Moderate-stable BMLs (*n* = 408)	Rapid-rise BMLs (*n* = 47)	*P*
Age	60.89 (8.97)	60.05 (9.05)	60.36 (8.39)	61.28 (8.99)	0.310
Sex (%)					
Male	233 (37.9)	54 (34.0)	15 (31.9)	164 (40.2)	0.263
Female	381 (62.1)	105 (66.0)	32 (68.1)	244 (59.8)	
Height	168.14 (94.05)	167.48 (91.91)	167.56 (79.47)	168.47 (96.43)	0.486
Weight	80.21 (15.55)	77.42 (15.22)	80.07 (16.18)	81.32 (15.51)	0.027
BMI	28.28 (4.41)	27.51 (4.52)	28.57 (4.61)	28.55 (4.31)	0.037
Income (%)					
≤ 50 k	198 (34.7)	58 (39.5)	14 (32.6)	126 (33.2)	0.377
> 50 K	372 (65.3)	89 (60.5)	29 (67.4)	254 (66.8)	
Marital status (%)					
Married	432 (71.1)	109 (68.6)	34 (73.9)	289 (71.7)	0.687
Unmarried/widowed/divorced	176 (28.9)	50 (31.4)	12 (26.1)	114 (28.3)	
Education level (%)					
Less than or equal to high school	86 (14.1)	20 (12.6)	4 (8.7)	62 (15.4)	0.742
Some college or college graduate	261 (42.9)	69 (43.4)	21 (45.7)	171 (42.4)	
Some graduate school or graduate degree	261 (42.9)	70 (44.0)	21 (45.7)	170 (42.2)	
Race (%)					
White	537 (87.5)	145 (91.2)	40 (85.1)	352 (86.3)	0.589
Black or African Americans	65 (10.6)	12 (7.5)	6 (12.8)	47 (11.5)	
Other	12 (2.0)	2 (1.3)	1 (2.1)	9 (2.2)	
Alcohol in typical week (%)					
None	106 (17.4)	24 (15.1)	5 (10.9)	77 (19.1)	0.351
< 1/week	212 (34.9)	64 (40.3)	15 (32.6)	133 (33.0)	
1–7/week	196 (32.2)	45 (28.3)	16 (34.8)	135 (33.5)	
> 7/week	94 (15.5)	26 (16.4)	10 (21.7)	58 (14.4)	
Smoking status (%)					
Never	305 (50.3)	83 (52.5)	19 (42.2)	203 (50.4)	0.367
Current	30 (5.0)	4 (2.5)	2 (4.4)	24 (6.0)	
Former	271 (44.7)	71 (44.9)	24 (53.3)	176 (43.7)	
Physical activity	169.35 (83.77)	166.87 (79.29)	159.64 (82.63)	171.44 (85.67)	0.600
History of knee injury (%)	150 (24.9)	38 (24.2)	11 (23.9)	101 (25.2)	0.956
History of knee surgery (%)	41 (6.7)	11 (6.9)	2 (4.4)	28 (6.9)	0.819

*BMI* body mass index, *BMLs* bone marrow lesions, *IQR* interquartile range

### Trajectories of BMLs identified by LCMMs

Evaluations based on the AIC, BIC, and average PP indicated that one group had a sample size of only 2.1% in four clusters, which could affect the stability and reliability of the model (Supplementary Table 1). Therefore, to ensure the accuracy and effectiveness of the analysis, we decided to select three groups for further analysis.

**Fig. 1:**
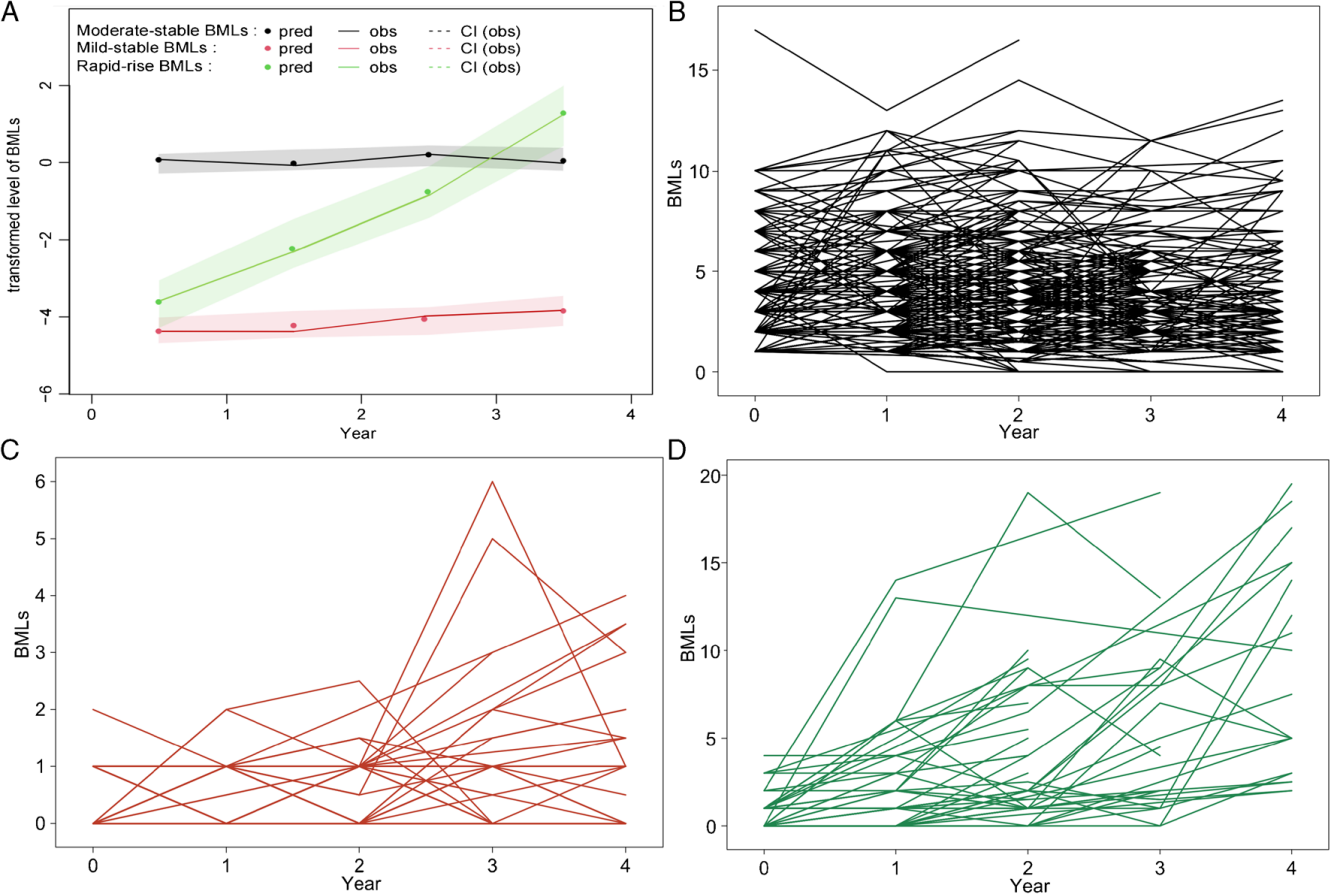
Trajectories (**A**) and individual trajectories (**B**). Moderate-stable BMLs; **C** Mild-stable BMLs; and **D** Rapid-rise BMLs of BMLs by the MRI Osteoarthritis Knee Score (MOAKS) scoring method

Three distinct BML trajectories were identified (Fig. 1): “Mild-stable BMLs” (*n* = 159, 25.9%), “‘Moderate-stable BMLs” (*n* = 408, 66.4%), and “Rapid-rise BMLs” (*n* = 47, 7.7%). All trajectories had a high mean PP (Mild-stable BMLs, 86%; Moderate-stable BMLs, 81%; and Rapid-rise BMLs, 77%), indicating a good model fit (Supplementary Table 1).

Baseline characteristics of the three BML trajectories were generally comparable, except that participants in the “Moderate-stable BMLs” and “Rapid-rise BMLs” trajectories had higher weights (Table 1).

### Predictors of BML trajectories

In multinomial logistic regression analyses, compared to the “Mild-stable BMLs” trajectory, current smokers were more likely to be in the “Moderate-stable BMLs” (OR = 2.089, 95% CI [1.719–2.540], *P* < 0.001) and “Rapid-rise” (OR = 2.462, 95% CI [2.079–2.915], *P* < 0.001) trajectories (Table 2). Female sex and meniscal tears were associated with an increased risk of being in the “Rapid-rise BMLs” trajectory (OR = 2.023, 95% CI [1.245–3.287], *P* = 0.004; OR = 2.504, 95% CI [2.169–2.890], *P* < 0.001). Participants who had higher education levels and drinking more alcohol were more likely to be in the “Rapid-rise BMLs” trajectory (OR = 1.849, 95% CI [1.247–2.742], *P* = 0.002; OR = 3.178, 95% CI [2.080–4.856], *P* < 0.001) and less likely to be in the “Moderate-stable BMLs” trajectory (OR = 0.674, 95% CI [0.519–0.876], *P* = 0.003; OR = 0.668, 95% CI [0.459–0.973], *P* = 0.036).

**Table 2:** Association between risk factors and BML trajectories

Covariates	Rapid-rise BMLs		Moderate-stable BMLs	
	*OR* (95% *CI*)	*P*	*OR* (95% *CI*)	*P*
Age	1.012 (0.969, 1.057)	0.586	1.044 (1.018, 1.07)	0.001
Sex (%)				
Male				
Female	2.023 (1.245, 3.287)	0.004	1.208 (0.788, 1.852)	0.387
Height	1.001 (0.998, 1.003)	0.535	1.001 (0.999, 1.002)	0.244
Weight	1.000 (0.969, 1.031)	0.981	1.018 (1.000, 1.035)	0.047
Changes in weight over 4 years	0.971 (0.904, 1.042)	0.409	0.964 (0.925, 1.005)	0.083
Income (%)				
≤ 50 k				
> 50 K	1.058 (0.612, 1.829)	0.841	1.482 (0.957, 2.297)	0.078
Marital status				
Married				
Unmarried/widowed/divorced	0.677 (0.411, 1.115)	0.126	0.873 (0.553, 1.376)	0.558
Education level				
Less than or equal to high school				
Some college or college graduate	1.624 (1.067, 2.473)	0.024	0.668 (0.503, 0.887)	0.005
Some graduate school or graduate degree	1.849 (1.247, 2.742)	0.002	0.674 (0.519, 0.876)	0.003
Race (%)				
White				
Black or African Americans	1.994 (1.555, 2.557)	< 0.001	1.677 (0.886, 3.176)	0.112
Other	2.544 (2.476–2.613)	< 0.001	1.736 (1.676, 1.797)	< 0.001
Alcohol in typical week (%)				
None				
< 1/week	1.836 (1.103, 3.058)	0.020	0.937 (0.631, 1.390)	0.746
1–7/weaak	2.374 (1.403, 4.017)	0.001	1.076 (0.732, 1.583)	0.708
> 7/week	3.178 (2.080, 4.856)	< 0.001	0.668 (0.459, 0.973)	0.036
Smoking status (%)				
Never				
Current	2.462 (2.079, 2.915)	< 0.001	2.089 (1.719, 2.540)	< 0.001
Former	1.152 (0.568, 2.336)	0.695	0.912 (0.596, 1.396)	0.672
History of knee injury				
No				
Yes	1.400 (0.621, 3.155)	0.418	1.381 (0.861, 2.216)	0.180
History of knee surgery				
No				
Yes	0.495 (0.387, 0.634)	< 0.001	0.674 (0.324, 1.402)	0.291
Physical activity	0.999 (0.994, 1.005)	0.755	1.001 (0.999, 1.004)	0.340
Comorb_score	1.421 (0.910, 2.219)	0.122	0.823 (0.605, 1.121)	0.217
JSN				
Grade 0				
Grade 1	1.201 (0.557, 2.592)	0.640	1.204 (0.773, 1.876)	0.411
Meniscal tears				
No				
Yes	2.504 (2.169, 2.890)	< 0.001	1.419 (0.863, 2.334)	0.168

*BML* bone marrow lesions, *OR* odds ratio, *CI* confidence interval. The reference group was the “Mild-stable BMLs” group

### Sensitive analysis

The results of the sensitivity analysis indicated that the trajectory changes within the subgroup of individuals with BML at baseline were similar to the overall population findings (Supplementary Fig. 1). Missing data on covariates ranged from 0.1 to 2.4%, and the associations between covariates and BML trajectories did not materially change after multiple imputations for missing data (Supplementary Table 2).

## Discussion

This is the first study to evaluate the trajectories of BMLs and their predictors. In this longitudinal study of middle-aged and older adults with or at increased risk of knee osteoarthritis OA, we identified three distinct BML trajectories over 4 years. Specifically, most participants had relatively stable BMLs, and few had enlarged BMLs. Moreover, we found that several baseline characteristics, including sex, race, smoking, alcohol consumption, education level, and meniscal tears may predict different BML trajectories.

While most of the previous studies have shown that BMLs can increase, decrease, or remain stable over time [9, 12, 17, 35, 36], our study failed to identify trajectory groups where BMLs decreased or fluctuated over 4 years, although such changes can be observed in several participants according to the individual trajectory plots (Fig. 1B–D). There are several potential reasons for the discrepancy. First, BMLs were calculated using the total MOKS scores from 15 subregions of the knee, and the semi-quantitative method for evaluating BMLs may not be sensitive enough compared to quantitative measurements. Thus, the increase and decrease of BMLs in different subregions can be counteracted, leading to a relatively stable BMLs over time. Second, despite being the first study that used the largest available data on repeat-measured BMLs, the sample size of this study was modest. Meanwhile, each trajectory group of BMLs was required to contain at least 5% of the total participants to ensure further data analysis, which may have limited trajectories with few participants being identified. Third, trajectory analyses are conducted at the population level and cannot fully reflect all individual trajectories, and our findings suggested that most participants had relatively stable or increased BMLs over 4 years. Therefore, larger size studies that measured BMLs with more sensitive methods are warrant to verify our findings.

There is little previous literature on risk factors of BMLs. In this study, participants with Moderate-stable BMLs were more likely to be older and have heavier weight compared with Mild-stable BMLs group. Previous studies have confirmed that age was one of the most important factors affecting the ability of the marrow to regenerate [37], leading to bone loss [38], bone fragility [39, 40], and eventually the development of BMLs. Moreover, aging and OA share several common characteristics, including the imbalance between reactive oxygen species production and the ability to repair tissue damages through endogenous antioxidant defenses. This may increase oxidative stress and further alter the bone microenvironment [41], which plays a key role in the formation of BMLs [42]. Additionally, a previous study also found that obesity was a risk factor of BMLs [43], and it is possible that this is due to biomechanical or metabolic mechanisms [44, 45].

We found that females were more likely to be in the Rapid-rise BMLs trajectory, but the underlying mechanisms on the relationship between sex and BMLs are unclear. OA is more prevalent in women and studies conducted exclusively in females have shown that BMLs were associated with the progression of cartilage defects [46, 47], suggesting that BMLs may play a role in the association between female sex and OA progression. Our study also showed that current smoking was associated with both Rapid-rise BMLs and Moderate-stable BMLs trajectories, compared to Mild-stable BMLs group. Previous studies suggested that smoking was associated with increased loss of knee cartilage and development of cartilage defects [48, 49], and it has also been found that smoking may impaire the ability of BMLs to resolve [50].

In this study, white people tended to have Mild-stable BMLs compared to other races, and high education levels and high frequency of alcohol consumption were associated with an increased risk of being in the Rapid-rise BMLs group and a decreased risk of being in the Moderate-stable BMLs group. The reasons for these associations are unknown, but it has been shown that race is associated with subchondral cysts and radiographic progression of OA [51, 52]. Importantly, we found that meniscal tears, but not history of knee injury, may lead to rapid-rise BMLs. This contrasts to previous studies suggesting an important role of knee injury in the progression of bone structural changes [14]. Moreover, patients who experienced knee surgery other than knee replacement were more likely to have mild-stable BMLs. Unlike previous studies that showed that physical activity may increase or decrease BMLs [53, 54], we did not find a significant association between physical activity and BML trajectories, and this is consistent with the findings of a cohort of healthy, community-based participants [12].

Strengths of this study included its long-term follow-up and the use of LCMM that allows for evaluating trajectories of BMLs over time. Limitations of the current study are worth noting. First, the assessment of BMLs was conducted annually, but it has been shown that BMLs can change over several weeks. Therefore, short-term variations of BMLs cannot be captured in this study. Second, while we selected common covariates according to previous literature to test for their roles in predicting BML trajectories, other unmeasured covariates, such as knee alignment, may also be important in change in BMLs. Third, since BMLs can enlarge and shrink over several weeks [55] but were measured annually in this study, we cannot be sure whether the BMLs observed in consecutive scans were the same lesions. However, a previous study has shown similar proportions of changes in BMLs at 6 months and 24 months [56], suggesting that BMLs are not highly variable over time. Lastly, our study relied on trajectory analysis, which necessitates the a priori assumption of specific trajectory patterns. Although we explored various possible trajectories and chose the models that best fit our data, this approach may still limit our understanding of the inherent data structure.

In conclusion, most participants had relatively stable BMLs, few had enlarged BMLs, and no trajectory of decreased BMLs was identified during the 4-year follow-up. Moreover, sociodemographic factors, lifestyle, and knee structural pathology play roles in predicting distinct BML trajectories.

### Supplementary Information

Below is the link to the electronic supplementary material.

Supplementary file1 (DOCX 10.7 MB)

## Data Availability

The datasets generated during and/or analyzed during the current study are available from the corresponding author on reasonable request.
