# Supplementary material for: Factors associated with trajectories of bone marrow lesions over 4 years: data from the Osteoarthritis Initiative
Source: Skeletal Radiol. 2024 Jan 20;53(7):1333–41. doi: 10.1007/s00256-024-04579-6 (PMC11093866; doi:10.1007/s00256-024-04579-6)
Supplement: Supplementary file 1 — Supplementary file1 (DOCX 10.7 MB) [file 256_2024_4579_MOESM1_ESM.docx]

## Supplementary Table 1. Fit statistics of LCMMs

| Link | G | loglik | npm | AIC | BIC | %class1 | %class2 | %class3 | %class4 | %class5 |
| --- | --- | --- | --- | --- | --- | --- | --- | --- | --- | --- |
| Linear | 1 | -4687.55 | 6 | 9387.10 | 9413.62 | 100.00 |  |  |  |  |
|  | 2 | -4561.14 | 9 | 9140.29 | 9180.07 | 3.42 | 96.58 |  |  |  |
|  | 3 | -4494.83 | 12 | 9013.67 | 9066.71 | 10.75 | 85.99 | 3.26 |  |  |
|  | 4 | -4468.01 | 15 | 8966.03 | 9032.33 | 5.05 | 8.96 | 83.88 | 2.12 |  |
|  | 5 | -4468.01 | 18 | 8972.03 | 9051.59 | 5.54 | 9.93 | 82.41 | 0.00 | 2.12 |
| Quadratic | 1 | -4138.85 | 8 | 8293.70 | 8329.06 | 100.00 |  |  |  |  |
|  | 2 | -4138.85 | 11 | 8299.70 | 8348.32 | 70.36 | 29.64 |  |  |  |
|  | 3 | -4042.58 | 14 | 8113.16 | 8175.04 | 26.71 | 67.59 | 5.70 |  |  |
|  | 4 | -4042.58 | 17 | 8119.15 | 8194.29 | 6.03 | 27.52 | 66.45 | 0.00 |  |
|  | 5 | -4042.58 | 20 | 8125.15 | 8213.55 | 6.19 | 30.29 | 63.52 | 0.00 | 0.00 |
| Beta | 1 | -3672.60 | 8 | 7361.20 | 7396.56 | 100.00 |  |  |  |  |
|  | 2 | -3637.62 | 11 | 7297.24 | 7345.86 | 68.24 | 31.76 |  |  |  |
|  | **3** | **-3606.89** | **14** | **7241.77** | **7303.65** | **66.45** | **25.90** | **7.65** |  |  |
|  | 4 | -3582.45 | 17 | 7198.90 | 7274.04 | 2.12 | 63.03 | 25.57 | 9.28 |  |
|  | 5 | -3565.08 | 20 | 7170.16 | 7258.56 | 1.63 | 63.03 | 7.98 | 24.27 | 3.09 |

Note: G: number of latent classes; loglik: log-likelihood of the model; npm: total number of parameters; AIC: Akaike information criterion; BIC: Bayesian information criterion;

## Supplementary Table 2. Association between risk factors and BML trajectories after multiple imputations for missing data

| Covariates | Rapid-rise BMLs | |  | Moderate-stable BMLs | |
| --- | --- | --- | --- | --- | --- |
|  | *OR* (95% *CI*) | *P* |  | *OR* (95% *CI*) | *P* |
| Age | 1.007 (0.970, 1.046) | 0.710 |  | 1.033 (1.010, 1.056) | 0.004 |
| Sex (%) |  |  |  |  |  |
| Male |  |  |  |  |  |
| Female | 1.368 (0.855, 2.187) | 0.191 |  | 0.958 (0.651, 1.409) | 0.827 |
| Height | 0.999 (0.996, 1.001) | 0.208 |  | 0.999 (0.998, 1.000) | 0.084 |
| Weight | 1.018 (0.990, 1.045) | 0.207 |  | 1.020 (1.004, 1.037) | 0.012 |
| Changes in weight over 4 years | 0.983 (0.921, 1.048) | 0.590 |  | 0.972 (0.936, 1.010) | 0.148 |
| Income (%) |  |  |  |  |  |
| <=50k |  |  |  |  |  |
| >50K | 1.484 (0.901, 2.444) | 0.121 |  | 1.644 (1.074, 2.518) | 0.022 |
| Marital status |  |  |  |  |  |
| Married |  |  |  |  |  |
| Unmarried/widowed/divorced | 0.839 (0.528, 1.334) | 0.459 |  | 0.945 (0.623, 1.434) | 0.791 |
| Education level |  |  |  |  |  |
| Less than or equal to high school |  |  |  |  |  |
| Some college or college graduate | 1.678 (1.138, 2.473) | 0.009 |  | 0.767 (0.441, 1.334) | 0.348 |
| Some graduate school or graduate degree | 1.631 (1.134, 2.347) | 0.008 |  | 0.721 (0.407, 1.276) | 0.262 |
| Race (%) |  |  |  |  |  |
| White |  |  |  |  |  |
| Black or African Americans | 2.169 (1.689, 2.787) | <0.001 |  | 1.737 (1.010, 2.987) | 0.046 |
| Other | 2.752 (2.676, 2.831) | <0.001 |  | 1.985 (1.855, 2.124) | <0.001 |
| Alcohol in typical week (%) |  |  |  |  |  |
| None |  |  |  |  |  |
| <1/week | 1.219 (0.777, 1.914) | 0.388 |  | 0.650 (0.465, 0.908) | 0.012 |
| 1-7/w | 1.880 (1.187, 2.977) | 0.007 |  | 0.951 (0.688, 1.316) | 0.763 |
| >7/w | 2.030 (1.410, 2.923) | <0.001 |  | 0.651 (0.477, 0.889) | 0.007 |
| Smoking status (%) |  |  |  |  |  |
| Never |  |  |  |  |  |
| Current | 2.132 (1.909, 2.381) | <0.001 |  | 3.220 (2.876, 3.606) | <0.001 |
| Former | 1.253 (0.667, 2.352) | 0.484 |  | 0.912 (0.620, 1.340) | 0.638 |
| History of knee injury |  |  |  |  |  |
| No |  |  |  |  |  |
| Yes | 1.209 (0.868, 1.684) | 0.261 |  | 1.264 (0.875, 1.827) | 0.212 |
| History of knee surgery |  |  |  |  |  |
| No |  |  |  |  |  |
| Yes | 0.512 (0.463, 0.566) | <0.001 |  | 0.905 (0.471, 1.740) | 0.765 |
| Physical activity | 0.999 (0.995, 1.003) | 0.656 |  | 1.001 (0.999, 1.004) | 0.364 |
| Comorb_score | 1.338 (0.882, 2.031) | 0.171 |  | 0.974 (0.739, 1.285) | 0.855 |
| JSN |  |  |  |  |  |
| Grade-0 |  |  |  |  |  |
| Grade-1 | 1.234 (0.636, 2.397) | 0.534 |  | 1.136 (0.761, 1.696) | 0.532 |
| Meniscal tears |  |  |  |  |  |
| No |  |  |  |  |  |
| Yes | 1.717 (1.464, 2.015) | <0.001 |  | 1.417 (0.920, 2.181) | 0.114 |

Note: BML: Bone Marrow Lesions; OR: odds ratio; CI: confidence interval.


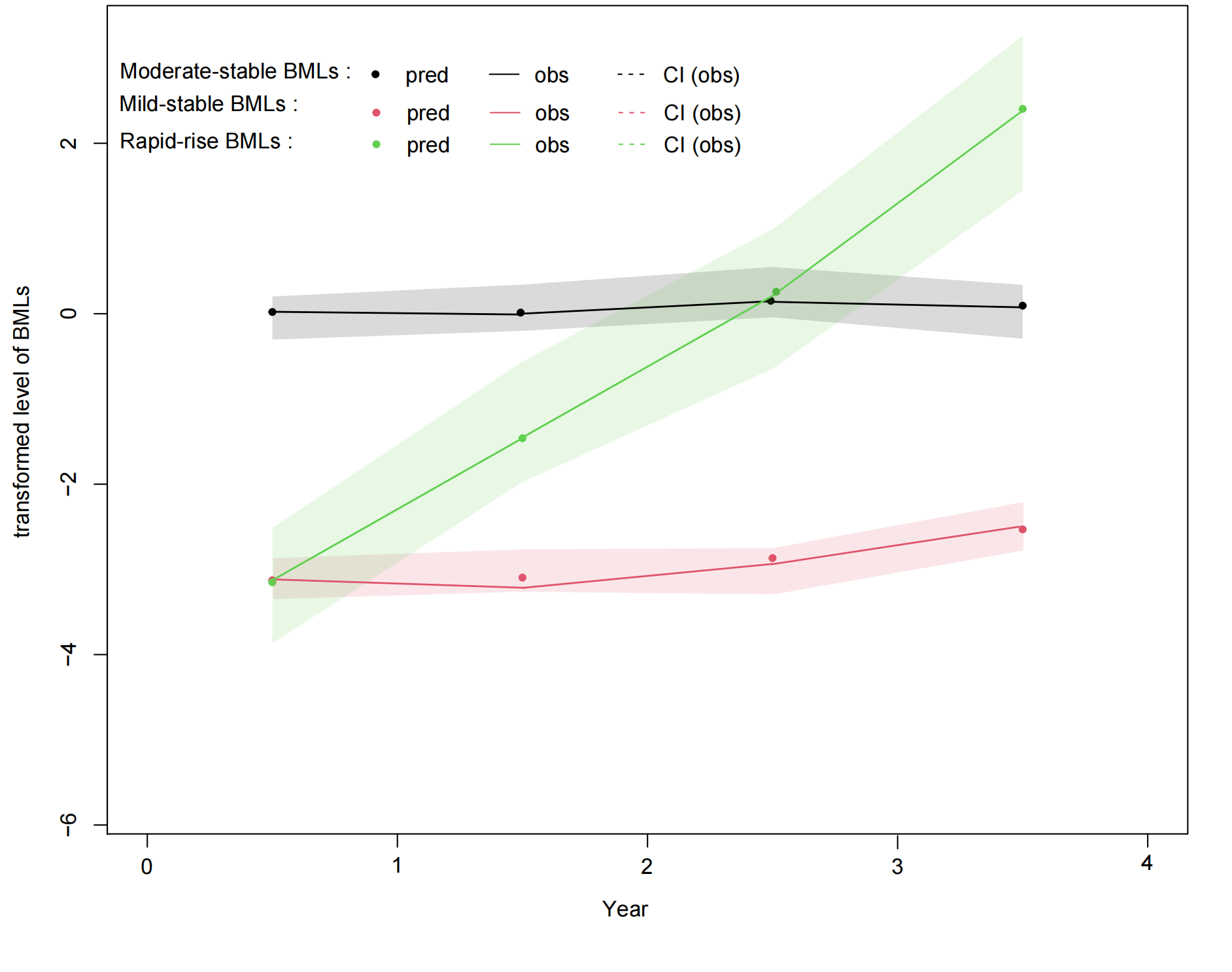


## Supplementary Figure 1 Trajectory of BMLs in patients with BML present at baseline
